# Overexpression of Notch3 and pS6 Is Associated with Poor Prognosis in Human Ovarian Epithelial Cancer

**DOI:** 10.1155/2016/5953498

**Published:** 2016-06-30

**Authors:** Zhaoxia Liu, Rongna Yun, Xiaolin Yu, Hui Hu, Genhua Huang, Buzhen Tan, Tingtao Chen

**Affiliations:** ^1^Department of Obstetrics & Gynecology, The Second Affiliated Hospital of Nanchang University, Nanchang, Jiangxi 330006, China; ^2^Department of Obstetrics & Gynecology, Nanshan Hospital, Shenzhen, Guangdong 518052, China; ^3^Department of Obstetrics & Gynecology, Xinxiang Central Hospital, Xinxiang, Henan 453600, China; ^4^Department of Obstetrics & Gynecology, The First Affiliated Hospital of Nanchang University, Nanchang, Jiangxi 330006, China; ^5^Institute of Translational Medicine, Nanchang University, Nanchang, Jiangxi 330031, China

## Abstract

Notch3 and pS6 play important roles in tumor angiogenesis. To assess the expression of Notch3 and pS6 in Chinese ovarian epithelial cancer patients, a ten-year follow-up study was performed in ovarian epithelial cancer tissues from 120 specimens of human ovarian epithelial cancer, 30 specimens from benign ovarian tumors, and 30 samples from healthy ovaries by immunohistochemistry. The results indicate that the expression of Notch3 and pS6 was higher in ovarian epithelial cancer than in normal ovary tissues and in benign ovarian tumor tissues (*p* < 0.01). In tumor tissues, Notch3 expression and pS6 expression were negatively associated with age (*p* > 0.05) but positively associated with clinical stage, pathological grading, histologic type, lymph node metastasis, and ascites (*p* < 0.05 or *p* < 0.01). A follow-up survey of 64 patients with ovarian epithelial cancer showed that patients with high Notch3 and pS6 expression had a shorter survival time (*p* < 0.01), in which the clinical stage (*p* < 0.05) and Notch3 expression (*p* < 0.01) played important roles. In conclusion, Notch3 and pS6 are significantly related to ovarian epithelial cancer development and prognosis, and their combination represents a potential biomarker and therapeutic target in ovarian tumor angiogenesis.

## 1. Introduction

Ovarian cancer represents one of the most aggressive neoplastic diseases in women, and 75% patients are diagnosed at advanced stage due to the lack of biomarkers for early diagnosis [1]. In 2012, ovarian cancer occurred in 239,000 women and caused 152,000 deaths worldwide and was more common in North America and Europe than in Africa and Asia [2]. Until now, the molecular etiology of this cancer has remained mostly unknown and therefore it is of great importance to explore the association of key proteins with poor prognosis in human ovarian epithelial cancer (the major histological type of ovarian cancer).

Notch signaling is a highly conserved cell-cell communication system present in multicellular organisms and has been characterized for its well-established role in a variety of physiological and pathological processes, including cancer development [3]. Notch3, a type of Notch receptor (Notch1, Notch2, Notch3, and Notch4), plays an important role in promoting ovarian tumorigenesis, cancer progression, and chemotherapy resistance via activating the PI3K/Akt/mTOR signaling pathway [4]. Ribosomal S6 kinase (S6K), a downstream effector of the PI3K/Akt pathway, is frequently activated in human ovarian cancer [5] and is significantly more prevalent in malignant tumors than in benign lesions. pS6 kinase is also involved in other aspects of cancer progression in addition to its well-established role in regulating proliferation and cell survival [5–7].

Although the roles of Notch3 and S6K in cancer development have been studied, no study has been carried out to combine the expression of Notch3 and S6K in relation to the prognosis of human ovarian epithelial cancer. It is known that Notch3 and S6K may complement their common functions in cancer development, but their roles in specific tumors are unique and context-dependent [8–11]. In the present study, we first investigated the expression of Notch3 and S6K in human ovarian epithelial cancer, to verify their expression related to clinicopathological features and prognosis in human ovarian epithelial cancer and to further evaluate their potential value as biological markers of aggressiveness in ovarian cancer, with the goal of improving the management of ovarian cancer patients.

## 2. Materials and Methods

### 2.1. Ethics Statement

Patient samples were obtained with written informed consent in accordance with ethics committee requirements at the participating institutes and the Declaration of Helsinki. Permission to carry out the study was obtained from the Institutional Review Board (IRB) of the Second Affiliated Hospital of Nanchang University.

### 2.2. Tissue Samples

Tissue samples were collected from 120 patients with ovarian epithelial cell carcinoma who underwent surgical resection at the Second Affiliated Hospital of Nanchang University between 1998 and 2008 (age range 36–68 years, median 49 years). All patients were histopathologically diagnosed based on clinical protocols, and none of them received presurgery chemotherapy or immunotherapy. Of the 120 patients, 41 patients (at stage I + II) underwent a hysterectomy + bilateral oophorectomy + omentum resection + appendectomy + pelvic lymph node dissection; 79 patients with advanced ovarian cancer (III + IV) underwent cytoreductive surgery, pelvic lymph node dissection, or pelvic lymph node biopsy; 37 patients had lymphatic metastasis and 70 patients had evident ascites. The histological results revealed that 77 patients had serous carcinoma and 43 patients had mucinous carcinoma; 17 tumors showed a high degree differentiation, 40 showed moderate differentiation, and 63 showed poor differentiation based on pathological grading.

In this study, 30 patients with benign ovarian cystadenoma (14 serous cystadenoma and 16 mucinous cystadenoma) were selected to perform a tumor stripping operation or unilateral salpingo-oophorectomy (age range 23–46 years, median 35 years). Another 30 patients (with either uterine fibroids, adenomyosis, or other nonovarian diseases) underwent hysterectomy + bilateral or unilateral oophorectomy and were selected as the control group (age range 46–69 years, median 58 years).

In the ovarian epithelial cell carcinoma group, 44 patients received combination chemotherapy of cisplatin + adriamycin + cyclophosphamide and 64 patients received carboplatin + paclitaxel. Twelve patients did not receive any postsurgery chemotherapy (see Table S1 in Supplementary Material available online at http://dx.doi.org/10.1155/2016/5953498).

### 2.3. Immunohistochemistry

Each tissue was fixed in formalin and embedded in paraffin and then sectioned and mounted on glass slides. After dewaxing in xylene and dehydration in graded alcohol, endogenous peroxidase activity was blocked with 3% hydrogen peroxide for 10 min. Then, the sections were subjected to antigen retrieval in a microwave oven at 700 W for 20 min in 10 mol/L citrate buffer solution (pH 6.0). After that, 10% goat serum albumin was applied for 20 min. Overnight incubation was carried out at 4°C with the following primary antibodies: rabbit polyclonal Notch3 (1 : 50 dilution; Santa Cruz Biotechnology, Santa Cruz, CA, USA) and p70S6k (1 : 50 dilution; Cell Signaling Technology, Beverly, MA, USA). Then, sections were incubated with the appropriate secondary antibodies at room temperature for 60 min and washed in phosphate-buffered saline (PBS). Diaminobenzidine (DAB) was used as the chromogen, and the sections were counterstained with hematoxylin. Samples incubated with PBS instead of primary antibodies were used as negative controls [12].

### 2.4. Evaluation of Immunostaining

All stained sections were evaluated and scored independently by two pathologists with no prior knowledge of the clinicopathological outcomes of the patients. The mean percentage of positive cells was scored as 0 (0%), 1 (1–25%), 2 (26–50%), 3 (51–75%), or 4 (76–100%). The staining intensity was scored as 0 (negative), 1 (weak), 2 (moderate), or 3 (strong). Final histological (*h*) scores were obtained for each case by multiplying the percentage and the intensity score. Protein expression levels were further analyzed by classifying *h* values as negative (−): 0-1, positive (+): 2–4, or strongly positive (++): 5–7 [12].

### 2.5. Statistical Analysis

SPSS 19.0 software was used for the statistical analysis. The significance of the relationships between Notch3 and pS6 expression and clinicopathological parameters was evaluated using the Wilcoxon and Kruskal-Wallis tests and Spearman's rank correlation. Survival rates were calculated using the Kaplan-Meier method and compared by the log-rank test. Multivariate analysis was used to identify independent prognostic factors for survival rates using the Cox proportional hazards regression model. *p* values < 0.05 were considered statistically significant [11–13].

## 3. Results

### 3.1. Expression of Notch3 and pS6 in Different Ovarian Tissues

**Figure 1:**
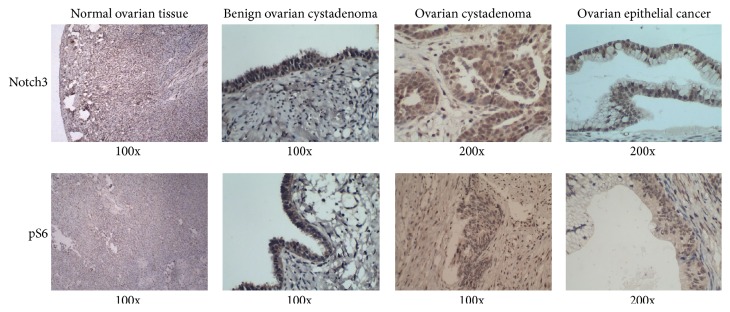
Evaluation of the protein expression of Notch3 and pS6 in normal ovarian tissue, ovarian cystadenoma, and ovarian epithelial cancer using immunohistochemistry.

The immunohistochemistry results show that Notch3 was mainly expressed in the cytoplasm and/or nucleus of ovarian epithelial cancer cells, while pS6 was mainly expressed in the cytoplasm (data not shown). In Figure 1 and Table 1, Notch3 protein was detected in normal ovarian tissue, ovarian cystadenoma, and ovarian epithelial cancer at different level. The positive expression rates of Notch3 in normal ovarian tissue, ovarian cystadenoma, and ovarian epithelial cancer were 16.7% (5/30), 70.0% (21/30), and 91.7% (110/120), respectively. Notch3 expression in ovarian epithelial cancer was significantly higher than in normal ovarian tissue (*p* < 0.01) and ovarian cystadenoma (*p* < 0.01), and Notch3 expression in ovarian cystadenoma was much higher than in normal ovarian tissue (*p* < 0.01).

Similar to Notch3 expression, the expression of pS6 in ovarian epithelial cancer (108/120, 90%) was significantly higher than in normal ovarian tissue (5/30, 16.7%) (*p* < 0.01) and ovarian cystadenoma (23/30, 76.7%) (*p* < 0.01), and a significant increase in pS6 was observed in ovarian cystadenoma compared to normal ovarian tissue (*p* < 0.01).

### 3.2. Correlation between the Clinicopathological Features and Expression of Notch3 and pS6

The relationship between ovarian epithelial cancer clinical stage and signaling molecule expression (Notch3 and pS6) was analyzed in Table 2. We found that Notch3 expression and pS6 expression were negatively associated with age (*p* > 0.05) but were positively associated with clinical stage, pathological grading, histological type, lymph node metastasis, and ascites. As shown in Table 2, Notch3 expression and pS6 expression were higher in stage III-IV than in stage I-II (*p* < 0.01, *p* < 0.01); similarly, Notch3 expression and pS6 expression were stronger with higher pathological grading compared to low pathological grading (*p* < 0.01, *p* < 0.01). The expression of Notch3 and pS6 was higher in serous cystadenocarcinoma, lymph node metastasis, and ascites than in mucinous cystadenocarcinoma (*p* < 0.01, *p* < 0.05) and in the absence of lymph node metastasis (*p* < 0.01, *p* < 0.01) and ascites (*p* < 0.01, *p* < 0.01).

The correlation analysis of Notch3 expression and pS6 indicated a positive correlation between these two proteins in ovarian epithelial cancer (*r*_*s*_ = 0.668, *p* < 0.01) (Tables 3 and 4).

### 3.3. Survival Analysis of Notch3 and pS6 Expression

A follow-up survey was performed on 64 patients with ovarian epithelial cancer who had received chemotherapy (carboplatin and paclitaxel) after surgery. Of these 64 patients, 46 patients died and 18 patients were censored or truncated. The shortest and longest survival times for these patients were 1 month and 102 months (with an average of 35.16 months), and the accumulated 1- to 5-year survival rates of the patients were 0.55, 0.36, 0.36, 0.28, and 0.21, respectively (Figure S1).

Based on Notch3 and pS6 protein expression, the 64 patients were divided into three groups: low Notch3 and pS6 expression (− −, *n* = 18), moderate Notch3 and pS6 expression (+− or −+, *n* = 4), and high Notch3 and pS6 expression (++, *n* = 42). As shown in Table 4, the overall survival of patients with low Notch3 and pS6 expression was longer (81.9 months), while the groups with high and moderate Notch3 and pS6 expression had a shorter survival time of 12.3 months and 16.8 months, respectively (*χ* ^2^ = 41.479, *p* < 0.01).

### 3.4. Multiple Factor Cox Regression Analysis of the Survival Rate

Table 5 shows the results of multiple factor Cox regression analysis of the survival rates of the 64 patients. When the analysis was performed, the survival time and dead/alive ratio were used as dependent variables, while age (<50 years = 1, ≥50 years = 2), clinical stage (stage I-II = 1, stage III-IV = 2), pathological grading (G1 = 1, G2 = 2, G3 = 3), histologic type (serous cystadenocarcinoma = 1, mucinous cystadenocarcinoma = 2), lymph node metastasis (yes = 1, no = 2), ascites (yes = 1, no = 2), Notch3 expression (negative staining = 0, positive staining = 1, strongly positive staining = 2), and pS6 expression (negative staining = 0, positive staining = 1, and strongly positive staining = 2) were the independent variables; the level of the variate was 0.05.

Among all the clinicopathological features, clinical stage III-IV was found to be a significant indicator of poor overall survival (hazard ratio (HR), 5.398; 95% confidence interval (CI) 1.154–25.259; *p* = 0.032) compared with stage I-II. Moreover, the Notch3 expression was also significantly associated with poor overall survival in these patients (HR, 8.362; 95% CI 2.154–32.461; *p* = 0.002) (Table 5).

As ascites is a key finding in cancer, we analyzed the relationship between the coexpression of Notch3 and pS6 expression and the presence of ascites. Higher expression of Notch3 and pS6 was associated with a higher positive rate of ascites (Table 6); the positive rates of ascites in patients with high, moderate, and low expression of Notch3 and pS6 were 82.1%, 51.9%, and 27.0%, respectively. The *χ* ^2^ test indicated that the expression level of Notch3 and pS6 has a significant positive correlation with ascites in these groups (*χ* ^2^ = 28.448, *p* < 0.01).

## 4. Discussion

The Notch signaling cascade is critical for cell proliferation, differentiation, development, and homeostasis [13], and deregulated Notch signaling is found in various diseases (e.g., T-cell leukemia, breast cancer, prostate cancer, colorectal cancer and lung cancer, and central nervous system malignancies) [14]. However, the mechanism of its regulation in ovarian cancer is unclear.

In our study, Notch3 expression in ovarian epithelial cancer was significantly higher than in benign cystadenoma and normal ovarian tissues (*p* < 0.01, Table 1) and was associated with clinical stage, pathological grading, histologic type, lymph node metastasis, and ascites (*p* < 0.01 or *p* < 0.05), suggesting that the Notch signaling pathway is in an activated state and probably plays an important role in the development of ovarian epithelial cancer [1, 8, 12, 13, 15, 16].

It is known that cancer occurrence is a comprehensive consequence of disorders in multiple signaling transduction pathways [9]. It has been shown that the PI3K/AKT signaling pathway is the key downstream mediator of Notch signaling; when Notch ligands activate the Notch signaling pathway, mTOR activates the downstream effectors S6k and eukaryotic translation initiator 4E binding protein 1 (4EBP1). Activated S6K phosphorylates the ribosomal protein pS6 and enhances the synthesis of the translation regulator p4EBP1 to regulate protein synthesis [5, 17, 18]. Therefore, Notch3 expression and pS6 expression play important roles in PI3K/AKT/mTOR signaling and ovarian epithelial cancer development. Our data also indicate a strong positive correlation between Notch3 expression and pS6 expression (*r*_*s*_ = 0.668, *p* < 0.01; Table 3).

In our follow-up survey of 64 patients with ovarian epithelial cancer (Table 4), the patients with high Notch3 and pS6 expression only survived for an average of 12.3 months, while patients with moderate and low Notch3 and pS6 expression survived for 16.8 months and 81.9 months, respectively (*χ* ^2^ = 41.479, *p* < 0.01). The clinical stage (*p* < 0.05) and Notch3 expression (*p* < 0.01) were more important than other clinicopathological features (Table 5). In addition, the occurrence of ascites in patients with a high level of Notch3 and pS6 expression was significantly higher than in the other groups, suggesting that a high level of Notch3 and pS6 expression may be associated with peritoneal implantation and spreading (Table 6).

In summary, although some studies have indicated that Notch3 or pS6 alone could be used as indicator of cancer development and prognosis [5, 11, 19], our results indicate that Notch3 and pS6 together have a strong relationship with the clinicopathological features of ovarian epithelial cancer and overall patient survival. However, Notch3 is not the only protein upstream of PI3K/AKT/mTOR signaling, and pS6 is not the only effector of PI3K/AKT/mTOR signaling [20, 21]. Moreover, the association analysis of Notch3 and pS6 (Table 3) indicated that five pS6 negative patients expressed moderate levels of Notch3 (4.2%), and three Notch3 negative patients expressed moderate levels of pS6 (2.5%). Therefore, the combined assessment of Notch3 and pS6 expression is a better choice of prognostic biomarker for overall survival in ovarian epithelial cancer than Notch3 or pS6 alone.

## Supplementary Material

The Fig. 1 S showed that 64 patients received carboplatin + paclitaxel chemotherapy, and the shortest survival time was 1 month and the longest survival time was 102 months, with an average survival time of 35.16 months, and the 1～5 survival rate were 0.55, 0.36, 0.36, 0.28, 0.21.

## Figures and Tables

**Table 1:** The protein expression of Notch3 and pS6 in normal ovarian tissue, ovarian cystadenoma, and ovarian epithelial cancer.

Characteristics	Cases, *n*	Notch3 expression, *n*			*p* value	pS6 expression, *n*			*p* value
		−	+	++		−	+	++	
Normal ovarian tissue	30	25	5	0	0.000	25	5	0	0.000
Ovarian cystadenoma	30	9	15	6		7	17	6	
Ovarian epithelial cancer	120	10	30	80		12	49	59	

**Table 2:** Correlation between the protein expression of Notch3 and pS6 proteins and clinicopathological parameters in patients with ovarian epithelial cancer.

Characteristics	Cases, *n*	Notch3 expression, *n*			*p* value	pS6 expression, *n*			*p* value
		−	+	++		−	+	++	
Age, yrs									
<50	79	7	19	53	0.947	6	37	36	0.558
≥50	41	3	11	27		6	12	23	

Clinical stages									
I~II	41	8	17	16	0.000	7	22	12	0.001
III~IV	79	2	13	64		5	27	47	

Pathological grading									
G1	17	7	7	3	0.000	5	7	5	0.001
G2	40	3	17	20		6	22	12	
G3	63	0	6	57		1	20	42	

Histologic type									
Serous cystadenocarcinoma	77	1	19	57	0.006	3	31	43	0.011
Mucinous cystadenocarcinoma	43	9	11	23		9	18	16	

Lymph node metastasis									
Yes	37	0	5	32	0.001	0	5	32	0.000
No	83	10	25	48		12	44	27	

Ascites									
Yes	70	2	9	59	0.000	2	21	47	0.000
No	50	8	21	21		10	28	12	

**Table 3:** Association between the expression of Notch3 and pS6.

Notch3	pS6				*r*_*s*_	*p*
	−	+	++	Total		
−	7	3	0	10	0.668	0.000
+	5	22	3	30		
++	0	24	56	80		

Total	12	49	59	120		

**Table 4:** The survival distribution of patients with different Notch3 and pS6 expression.

Notch3 and pS6	Average survival (month)	Overall survival		
		95% CI	*χ* ^2^	*p*
−−	81.916 ± 6.541	69.095–94.737	41.479	<0.01
−+ or +−	16.750 ± 2.136	12.563–20.937		
++	12.338 ± 1.947	8.521–16.155		

Total	35.162 ± 4.985	25.391–44.932		

**Table 5:** Multiple COX regression analysis of patients with ovarian epithelial cancer.

Variable	*B*	SE	Wald	*p*	HR	95% CI
Clinical stages	1.686	0.787	4.586	0.032	5.398	1.154–25.259
Notch3	2.124	0.692	9.418	0.002	8.362	2.154–32.461

**Table 6:** Relationship between the coexpression of Notch3 and pS6 expression and ascites.

Notch3 and pS6	No ascites		Ascites		Total	*χ* ^2^	*p*
	*n*	%	*n*	%			
−−	27	73.0	10	27.0	37	28.448	0.000
−+ or +−	13	48.1	14	51.9	27		
++	10	17.9	46	82.1	56		

Total	50	41.7	70	58.3	120		
