# Supplementary material for: Overexpression of Notch3 and pS6 Is Associated with Poor Prognosis in Human Ovarian Epithelial Cancer
Source: Mediators Inflamm. 2016 Jun 30;2016:5953498. doi: 10.1155/2016/5953498 (PMC4944072; doi:10.1155/2016/5953498)
Supplement: Supplementary file 1 — The Fig. 1 S showed that 64 patients received carboplatin + paclitaxel chemotherapy, and the shortest survival time was 1 month and the longest survival time was 102 months, with an average survival time of 35.16 months, and the 1～5 survival rate were 0.55, 0.36, 0.36, 0.28, 0.21. [file 5953498.f1.pdf]

Table S1 Characteristics of the patients (n = 120).

Clinicopathological features	Cases
Total No. of patients	120#
Histological type	
Serous adenocarcimoma	77
Mucinous adenocarcimoma	43
Clinical stages	
I-II	41##
III-IV	79###
Lymph node metastasis	
Yes	37
No	83
Ascites	
Yes	70
No	50
Adjuvant chemotherapy	
Yes	108####
No	12

Note: #, the median age is 49 ± 6 years old (ranged from 36 to 68). ##, received hysterectomy plus bilateral oophorectomy, omentum resection, appendectomy and pelvic lymph node dissection. ###, cytoreductive surgery. ####, 44 received Cisplatin + Adriamycin + Cyclophosphamide, and 64 received carboplatin +Taxol.

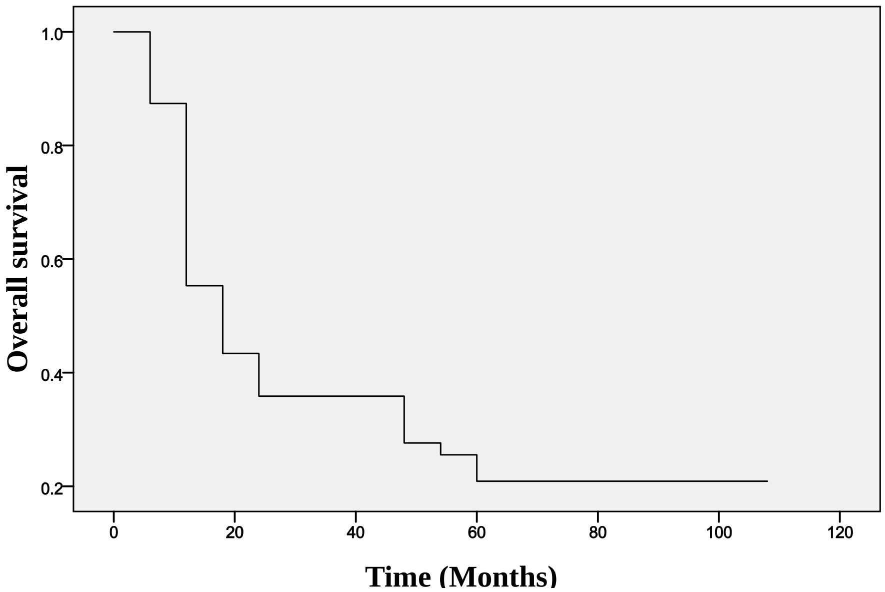

Fig. S1 The survival distribution function of 64 ovarian epithelial cancer patients on followed-up
